# Clonal Hematopoietic Mutations in Plasma Cell Disorders: Clinical Subgroups and Shared Pathogenesis

**DOI:** 10.1093/gpbjnl/qzaf027

**Published:** 2025-03-27

**Authors:** Xuezhu Wang, Liping Zuo, Yanying Yu, Xinyi Xiong, Jian Xu, Bing Qiao, Jia Chen, Hao Cai, Qi Yan, Hongxiao Han, Xin-xin Cao, Jun Deng, Chunyan Sun, Jian Li

**Affiliations:** Department of Hematology, Peking Union Medical College Hospital, Chinese Academy of Medical Sciences and Peking Union Medical College, Beijing 100730, China; Institute of Hematology, Union Hospital, Tongji Medical College, Huazhong University of Science and Technology, Wuhan 430022, China; Department of Hematology, Peking Union Medical College Hospital, Chinese Academy of Medical Sciences and Peking Union Medical College, Beijing 100730, China; Department of Hematology, Peking Union Medical College Hospital, Chinese Academy of Medical Sciences and Peking Union Medical College, Beijing 100730, China; Institute of Hematology, Union Hospital, Tongji Medical College, Huazhong University of Science and Technology, Wuhan 430022, China; Institute of Hematology, Union Hospital, Tongji Medical College, Huazhong University of Science and Technology, Wuhan 430022, China; Department of Hematology, Peking Union Medical College Hospital, Chinese Academy of Medical Sciences and Peking Union Medical College, Beijing 100730, China; Department of Hematology, Peking Union Medical College Hospital, Chinese Academy of Medical Sciences and Peking Union Medical College, Beijing 100730, China; School of Life Sciences, Tsinghua University, Beijing 100084, China; Department of Hematology, Peking Union Medical College Hospital, Chinese Academy of Medical Sciences and Peking Union Medical College, Beijing 100730, China; Department of Hematology, Peking Union Medical College Hospital, Chinese Academy of Medical Sciences and Peking Union Medical College, Beijing 100730, China; Institute of Hematology, Union Hospital, Tongji Medical College, Huazhong University of Science and Technology, Wuhan 430022, China; Institute of Hematology, Union Hospital, Tongji Medical College, Huazhong University of Science and Technology, Wuhan 430022, China; Department of Hematology, Peking Union Medical College Hospital, Chinese Academy of Medical Sciences and Peking Union Medical College, Beijing 100730, China

**Keywords:** Multiple myeloma, Primary light-chain amyloidosis, POEMS syndrome, Monoclonal gammopathy of undetermined significance, Clonal hematopoiesis

## Abstract

Plasma cell disorders (PCDs) are marked by the clonal proliferation of abnormal plasma cells and bone marrow plasma cells (BMPCs), causing various clinical complications. These PCDs include subtypes with distinct clinical features. Multiple myeloma (MM) and monoclonal gammopathy of undetermined significance (MGUS) are more common and relatively well-studied. In contrast, primary light-chain amyloidosis (AL) and POEMS syndrome (POEMS) are rare and remain less understood. To investigate the role of clonal hematopoietic (CH) mutations and potential interconnections in these diseases, we sequenced CH mutations in lymphoid and myeloid lineages, as well as myeloma driver gene mutations, in BMPCs from affected patients. Recurrent lymphoid CH mutations (in *FAT1*, *KMT2D*, *MGA*, and *SYNE1*) and myeloma driver gene mutations (in *ZFHX3* and *DIS3*) were found in the dominant clonal and subclonal plasma cell populations. These moderately aging-associated lymphoid CH mutations had a higher burden in MM than in AL or POEMS. Binary matrix factorization of these mutations revealed the subgroups associated with progression-free survival (PFS) (observed in MM, AL, and POEMS), age at diagnosis (in AL and POEMS), serum differential free light chain (dFLC) levels, plasma cell burden (in AL), and serum vascular endothelial growth factor (VEGF) levels (in POEMS). Moreover, the poor PFS associated with *MGA* or *SYNE1* mutations was confirmed across MM, AL, and POEMS. CH mutations partially explained the shared pathogenesis of MM, AL, POEMS, and MGUS, and helped identify patient subgroups with specific clinical features.

## Graphical abstract

**Graphical abstract:**
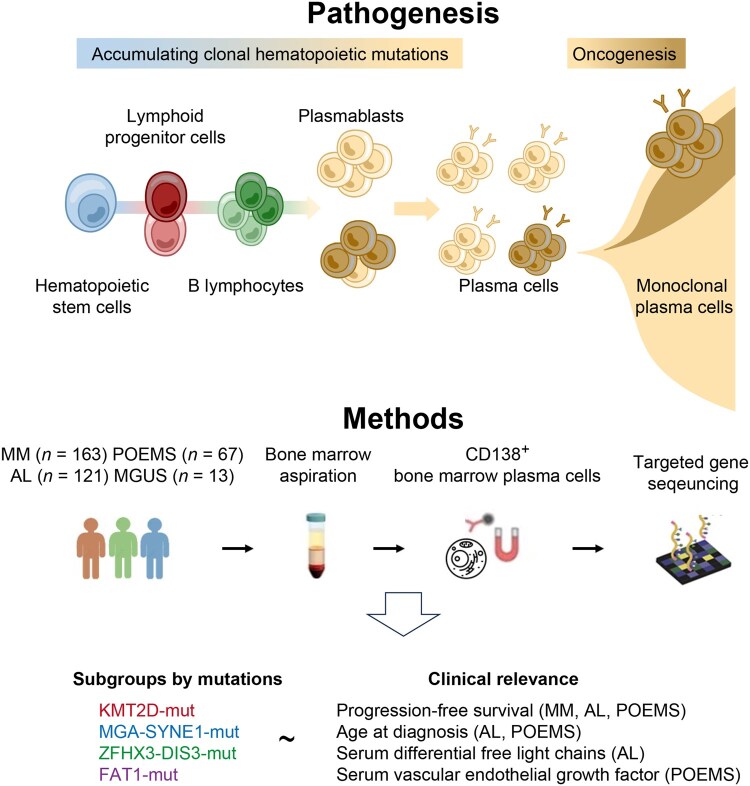

## Introduction

Plasma cell disorders (PCDs) represent a group of clonal hematopoietic (CH) diseases specifically affecting bone marrow plasma cells (BMPCs) [1]. These disorders are characterized by the abnormal expansion of plasma cells derived from a single clone and driven by mutations, leading to disruptions in the bone marrow environment and a variety of systemic diseases [2]. According to different clinical manifestations, PCDs are classified into more commonly observed ones: multiple myeloma (MM) and monoclonal gammopathy of undetermined significance (MGUS), as well as rare diseases: primary light-chain amyloidosis (AL) and POEMS syndrome (POEMS). The clinical presentations of these PCDs vary widely: MM commonly presents with osteolytic fractures [3]; MGUS is typically asymptomatic; AL leads to organ damage, such as nephrotic syndrome and restrictive cardiomyopathy, due to light chain deposits [4,5]; and POEMS includes polyneuropathy, organomegaly, endocrinopathy, and skin changes [6,7].

PCDs are considered genetic diseases, as mutations and cytogenetic abnormalities drive the dysregulated growth of BMPCs. The mutational pathogenesis of MM has been well-characterized and shown to be clinically significant by numerous independent studies [8–11]. A model of clonal evolution of MM plasma cells caused by primary (mostly cytogenetic abnormalities) and secondary (cytogenetic and single nucleotide variants) factors has been established, involving MGUS and smoldering MM (SMM) as the early premalignant stage of MM. However, MGUS is relatively common in older populations, with an occurrence rate of 3% among individuals over 50 years old due to undetermined mechanisms and significance [12].

AL and POEMS are the rare subtypes of PCDs that are not characterized by genomic instability and rapid cell proliferation. Due to limited observations and inconsistencies across studies, the mutational pathogenesis of AL and POEMS has remained uncertain for decades, and their study is often aligned with the understanding of MM and MGUS. For example, genome-wide association studies revealed the genetic loci implicating the common etiology in MM, AL, and MGUS [13] and the genetic susceptibility shared by MM and AL [14]. Next-generation sequencing methods enabled the profiling of mutations in AL and POEMS, and subsequently, consistent mutated genes were observed, which included *FAT4*, *IGLL5*, *ZFHX3*, *EP300*, *DIS3*, *KLHL6*, *KMT2B*, and *KRAS* in AL [15–18], and *HIST1H1B*, *POLE*, *KLHL6*, *RYR1*, and *USH2A* in POEMS [19,20]. A biological continuum was proposed based on findings that the classical myeloma driver mutation burden is higher in MM than in AL or MGUS [2].

Despite research into the genetics of AL and POEMS, the pathogenic mechanisms remain unclear. Although some studies have attempted to correlate genetic events with clinical features like overall survival [16] and serum vascular endothelial growth factor (VEGF) elevation [19], most studies have been limited by small sample sizes and analytical constraints. To date, only one study has identified the significant prognostic value for *HIST1H1E*, *ASCC3*, and *ASB15* in AL [16]. Interestingly, some mutations identified in AL and POEMS overlap with known age-related CH mutations, which are somatic mutations associated with a higher risk of blood malignancies, such as *FAT4*, *ZFHX*, *KLHL6*, and *RYR1* [15–20]. CH mutations have been associated with an increased risk of lymphoid or myeloid malignancies [21] and demonstrated to accumulate in aging blood cell lineages [22] and the diseased cells of other CH disorders [23]. Environmental stresses, such as metabolic disorders, cytotoxic agent exposure, and inflammatory stress, can induce DNA damage that promotes clonal selection and leads to the development of CH mutations [24]. Previous studies captured the CH mutations in peripheral blood cells and whole bone marrow, while how these mutations accumulate in the B cell lineage and, finally, the myeloma cells remains largely unexplored [25–28].

**Figure 1:**
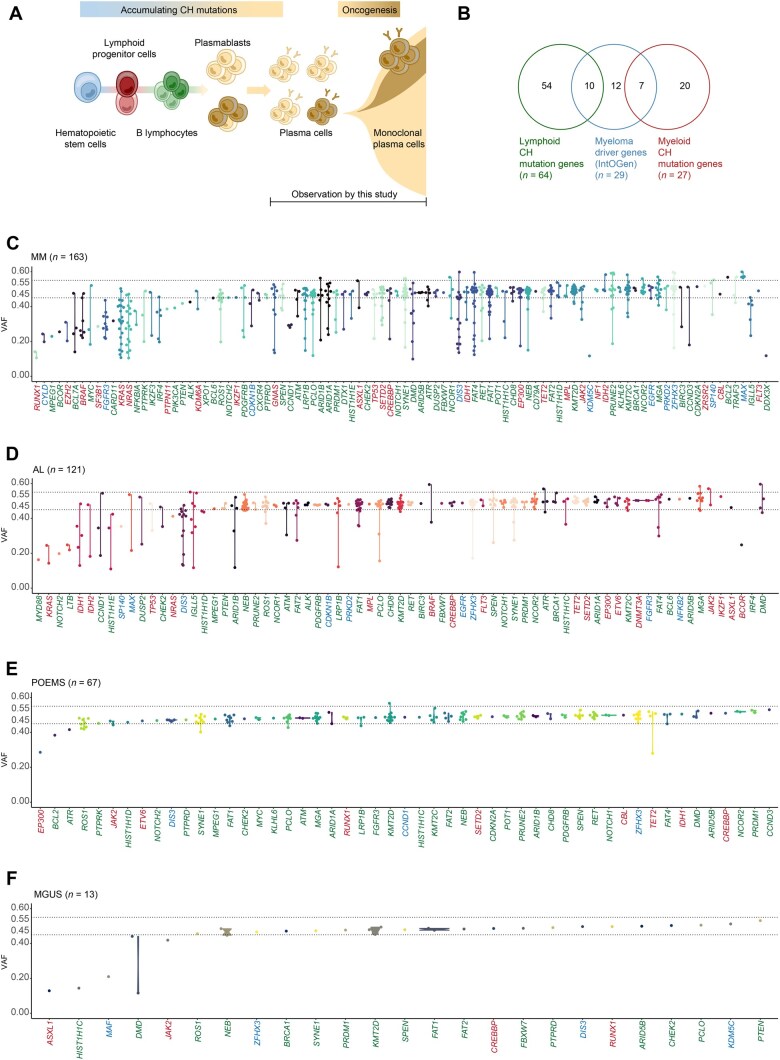
Lymphoid and myeloid CH mutation profiling in PCDs **A**. CH mutations and oncogenesis in PCDs. **B**. Venn diagram showing lymphoid and myeloid CH mutation genes and myeloma driver mutation genes. **C**.–**F**. VAF of mutations in BMPCs in MM (*n* = 163) (C), AL (*n* = 121) (D), POEMS (*n* = 67) (E), and MGUS (*n* = 13) (F). Green means lymphoid CH mutation genes, red means myeloid CH mutation genes, and blue means myeloma driver genes. The colors are randomly chosen in the same spectrum. CH, clonal hematopoietic; PCD, plasma cell disorder; BMPC, bone marrow plasma cell; MM, multiple myeloma; AL, primary light-chain amyloidosis; POEMS, POEMS syndrome; MGUS, monoclonal gammopathy of undetermined significance; VAF, variant allele frequency.

Here, we hypothesized that CH mutations captured in BMPCs might participate in the pathogenesis of PCDs and have relevance to clinical manifestations and clinical biology (**Figure 1**A). To profile the CH mutations in PCDs, we sequenced a total of 364 samples from patients with MM (*n* = 163), AL (*n* = 121), POEMS (*n* = 67), and MGUS (*n* = 13), using a targeted sequencing panel of 103 genes including lymphoid and myeloid CH mutation genes and myeloma driver genes. The novelty of our study is using a standard pipeline to sequence a large number of patients with rare PCDs (AL and POEMS) along with the relatively common PCDs (MM) for comparison. This dataset enables us to explore rare PCDs by leveraging prior knowledge of MM as a reference.

## Results

### Distribution of lymphoid and myeloid CH mutations in clonal BMPCs in PCDs

To investigate the role of CH mutations in PCD pathogenesis, targeted gene sequencing was performed on BMPCs from patients with MM (*n* = 163), AL (*n* = 121), POEMS (*n* = 67), and MGUS (*n* = 13). The median follow-up time of these cohorts was 12.8 months (range: 1.0–66.6 months) for MM, 23.1 months (range: 0.4–51.8 months) for AL, and 20.5 months (range: 3.0–42.8 months) for POEMS. We don’t have follow-up records of the patients with MGUS (**Table 1**).

**Table 1:** Clinical characteristics of the 364 patients with PCDs

	**MM** (*n* = 163)	**AL** (*n* = 121)	**POEMS** (*n* = 67)	**MGUS** (*n* = 13)
Male: number (%)	92 (56.4)	77 (63.6)	32 (47.8)	7 (53.8)
Age (year): median (range)	62.0 (38.0–82.0)	60.0 (39.0–85.0)	49.0 (27.0–70.0)	64.6 (43.0–85.0)
Percentage of BMPCs (%): median (range)	29.0 (1.0–95.0)	4.5 (0.5–25.0)	1.5 (0–6.5)	4.5 (1.0–7.5)
Follow-up time (month): median (range)	12.8 (1.0–66.6)	23.1 (0.4–51.8)	20.5 (3.0–42.8)	NA
**Cytogenetic abnormalities**				
t(11;14): number (%)	33 (20.2)	28 (23.1)	NA	NA
1q21(gain): number (%)	64 (39.3)	NA	NA	NA
17p(del): number (%)	19 (11.7)	NA	NA	NA
t(4;14): number (%)	29 (17.8)	NA	NA	NA
**Biological markers**				
Serum dFLC (mg/l): median (range)	NA	227.5 (5.5–57,486.3)	NA	NA
Serum VEGF (pg/ml): median (range)	NA	NA	4225 (200–16,389)	NA
**ISS for MM / Mayo 2012 stages for AL**				
Stage I: number (%)	28 (17.2)	26 (21.5)	NA	NA
Stage II: number (%)	61 (37.4)	36 (29.8)	NA	NA
Stage III: number (%)	74 (45.4)	25 (20.7)	NA	NA
Stage IV: number (%)	NA	34 (28.1)	NA	NA
**Most frequently used first-line therapies**				
VCD: number (%)	49 (30.1)	47 (38.8)	0	NA
VRD: number (%)	47 (28.8)	0	0	NA
RD: number (%)	0	0	37 (55.2)	NA

*Note*: PCD, plasma cell disorder; MM, multiple myeloma; AL, primary light-chain amyloidosis; POEMS, POEMS syndrome; MGUS, monoclonal gammopathy of undetermined significance; BMPC, bone marrow plasma cell; NA, not applicable; dFLC, differential free light chain; VEGF, vascular endothelial growth factor; VCD, bortezomib, cyclophosphamide, and dexamethasone; VRD, lenalidomide, bortezomib, and dexamethasone; RD, lenalidomide and dexamethasone; ISS, International Staging System.

In theory, CH mutations accumulate in the premalignant cell lineage and are enriched in the diseased and expanded monoclonal plasma cells (Figure 1A). The clonal architecture of BMPCs usually includes a dominant BMPC clone and multiple subclonal BMPCs. It is generally assumed that the dominant BMPC clones contain heterozygous CH mutations [0.45 < variant allele frequency (VAF) < 0.55] [29]. Likewise, according to the model of genomic evolution in MM, CH mutations in subclonal BMPCs should be driver mutations or mutations gained at late oncogenesis (VAF < 0.45) [8]. A collection of 64 lymphoid CH mutations, 27 myeloid CH mutations, and 29 myeloma driver gene mutations were sequenced according to the previous studies that defined these mutation categories in large-scale populations [21,30] (Figure 1B; Table S1).

In MM, myeloma driver genes such as *KRAS*, *NRAS*, *BRAF*, *MYC*, and *FGFR3* were mainly subclonal, whereas myeloid CH mutations, such as those in *TP53*, *CREBBP*, *EP300*, *TET2*, and *JAK2*, and lymphoid CH mutations, such as those in *MGA*, *SYNE1*, *FAT1*, *KMT2D*, and *HIST1H1D1*, appeared in dominant clonal BMPCs (Figure 1C; Table S2). Notably, *DIS3* mutations were abundant in both dominant clonal and subclonal BMPCs. In AL, relatively sparse subclonal variants were found in *MYD88*, *KRAS*, *NOTCH2*, *IDH1*, *IDH2*, *CCND1*, *HIST1H1E*, *DUSP2*, *TP53*, and *NRAS*. *DIS3* and *IGLL5* mutations were found in both dominant clonal and subclonal BMPCs (Figure 1D; Table S2). Most mutations in POEMS occurred in lymphoid and myeloid CH mutation genes, among which very rare subclonal ones were found in *EP300*, *BCL2*, and *ATR* (Figure 1E; Table S2). In MGUS, the known premalignant stage of MM, dominant lymphoid and myeloid CH mutations occurred in *ZFHX3*, *SYNE1*, *KMT2D*, *FAT1*, and *DIS3* (Figure 1F; Table S2). These findings indicate that CH mutations start to accumulate since the early stage of clonal BMPC formation, before the occurrence of driver mutations, and likely in both lymphoid and myeloid lineages.

Despite the distinct burden of subclonal mutations, some CH mutations were conservative among MM, AL, POEMS, and MGUS, including lymphoid and myeloid CH mutations, such as those in *KMT2D*, *FAT1*, *MGA*, and *SYNE1*, and myeloma driver genes like *ZFHX3* and *DIS3*. This overlap among the CH mutations in different PCDs prompted us to study its role as a common mechanism of PCD pathogenesis.

### Common and distinct CH mutations across PCD subtypes: variations in pathogenicity and mutation burden

**Figure 2:**
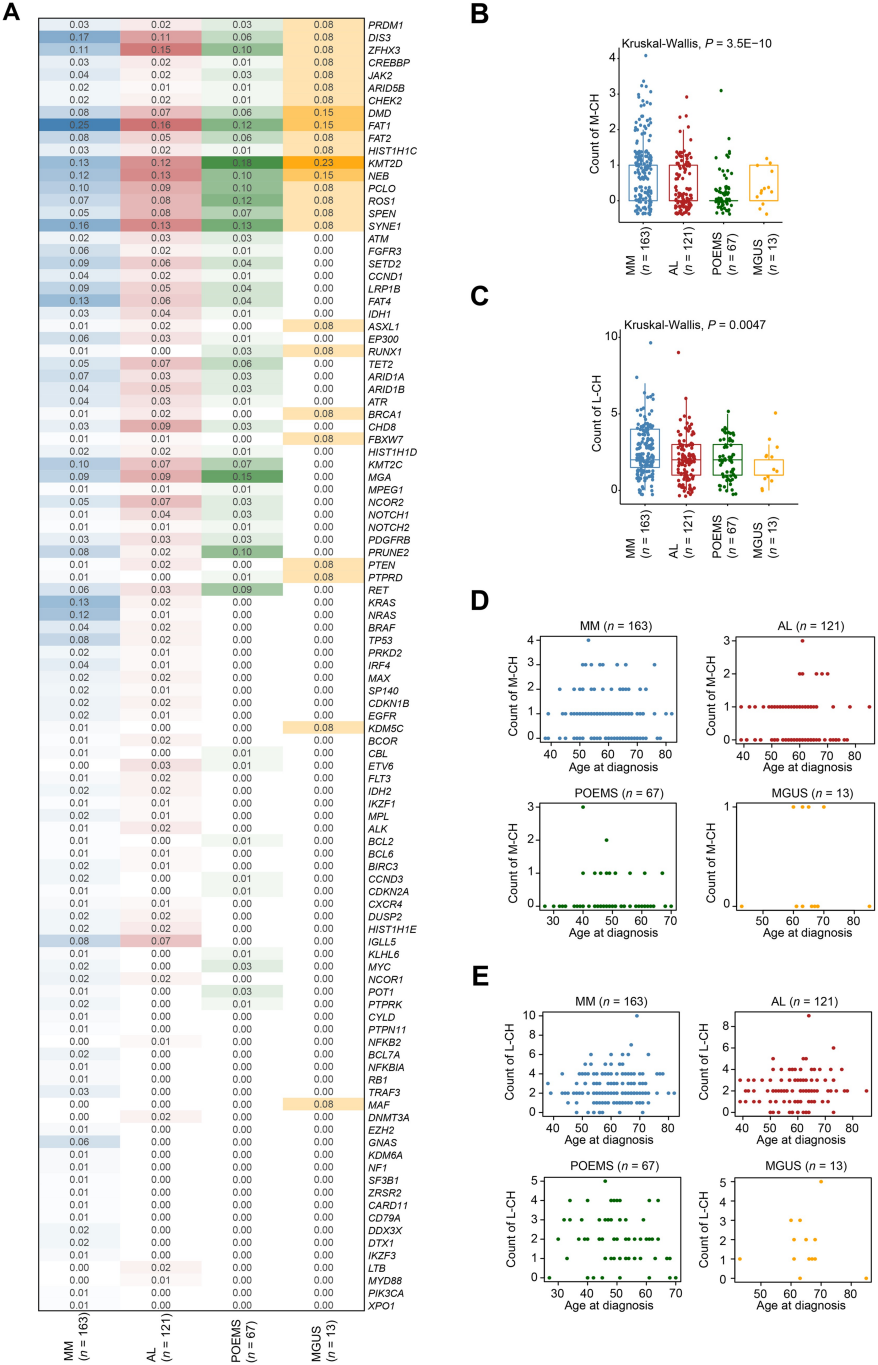
Comparative analysis of CH mutations in the four subtypes of PCDs **A**. Mutation frequency and co-occurrence of CH mutations in MM, AL, POEMS,and MGUS. **B**. Myeloid CH mutation burden in the four subtypes of PCDs. **C**. Lymphoid CH mutation burden in the four subtypes of PCDs. **D**. Association between patients’ age at diagnosis and myeloid CH mutation burden. **E**. Association between patients’ age at diagnosis and lymphoid CH mutation burden. M-CH, myeloid CH; L-CH, lymphoid CH.

We further investigated the different and common features of CH mutations across the four subtypes of PCDs. Some CH mutations appeared in all disease subtypes, including those in *PRDM1*, *DIS3*, *ZFHX3*, *CREBBP*, *JAK2*, *ARID5B*, *CHEK2*, *DMD*, *FAT1*, *FAT2*, *HIST1H1C*, *KMT2D*, *NEB*, *PCLO*, *ROS1*, *SPEN*, and *SYNE1*. In contrast, some CH mutations were more frequently observed in one subtype, such as the *MGA* mutation in POEMS (**Figure 2**A).

The mutational burden of myeloid (*P* = 3.5E−10) (Figure 2B) and lymphoid (*P* = 0.0047) CH mutations (Figure 2C) was similarly higher in MM than in AL or POEMS, with the lowest burden in MGUS. Given that the CH mutation burden in peripheral blood cells increases with an individual’s age [21], we plotted the correlation between the patient’s age at diagnosis and the frequency of lymphoid and myeloid CH mutations. As expected, a higher count of myeloid CH mutations was observed in patients with MM aged 50 to 70, patients with AL aged 60 to 70, and patients with POEMS aged 40 to 50 (Figure 2D). This suggests that the accumulation of myeloid CH mutations with age may vary across different diseases, potentially influenced by disease-specific mechanisms or biological factors. For the lymphoid CH mutations, an increased mutational burden was observed in patients aged 50 to 70 with MM, AL, and POEMS (Figure 2E), suggesting a more uniform age-dependent increase that might be less affected by disease-specific factors. Regrettably, the small size of the MGUS dataset could not support solid conclusions about the association between CH mutation burden and patient’s age at diagnosis.

### Binary matrix factorization analysis identified the subgroups of PCDs based on the profiling of CH mutations

**Figure 3:**
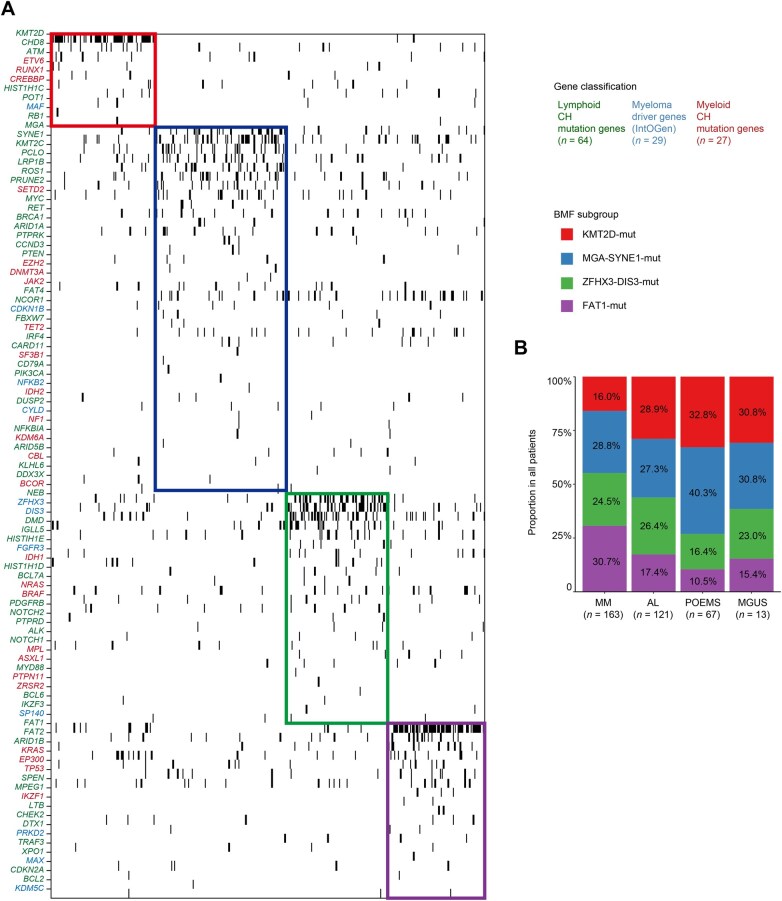
PCD subgroups based on BMF of CH mutations **A**. BMF of lymphoid and myeloid CH mutations and myeloma driver mutations (*k* = 4). Green means lymphoid CH mutation genes, red means myeloid CH mutation genes, and blue means myeloma driver genes. **B**. Proportions of the four subgroups in MM, AL, POEMS, and MGUS. Red means KMT2D-mut subgroup, blue means MGA-SYNE1-mut subgroup, green means ZFHX3-DIS3-mut subgroup, and purple means FAT1-mut subgroup. BMF, binary matrix factorization.

We performed binary matrix factorization (BMF), an algorithm that separates the pattern from the noise, on the CH mutations in PCDs, which generated four genetic subgroups: KMT2D-mut, MGA-SYNE1-mut, ZFHX3-mut, and FAT1-mut. We named these subgroups based on the most frequently occurring differential mutations within each group, ensuring concise and clear expression. The KMT2D-mut subgroup harbored variants in *KMT2D*, *CHD8*, *ATM*, *ETV6*, *RUNX1*, *CREBBP*, and *HIST1H1C*. The MGA-SYNE1-mut subgroup exhibited recurrent mutations in *SYNE1*, *KMT2C*, *MGA*, *LPR1B*, *SETD2*, and *DNMT3A*. The ZFHX3-mut subgroup included variants in *ZFHX3*, *DIS3*, *IGLL5*, *FGFR3*, *NRAS*, and *BRAF*. The FAT1-mut subgroup demonstrated high mutation rates in *FAT1*, *FAT2*, *ARID1B*, *KRAS*, *EP300*, and *TP53* (**Figure 3**A, Figure S1A–F). These BMF subgroups constituted MM, AL, POEMS, and MGUS in different proportions (Figure 3B), indicating a potential relation between BMF subgroups and clinical manifestations.

We also used a multivariate Cox proportional hazards model to calculate the hazard ratio and significance for each CH mutation as the prognostic factors of patients’ progression-free survival (PFS) (Table S3). This multivariable analysis helps to reveal the association between each mutation and patients’ survival with consideration of potential confounding factors. The results showed several CH mutations significantly associated with unfavorable prognosis in each PCD subtype, such as *NOTCH1* and *ZRSR2* in MM, *CREBBP* and *LTB* in AL, and *JAK2* and *CCND1* in POEMS (**Table 2**).

**Table 2:** Multivariate Cox proportional hazards model of the CH mutations

	Beta (**β**)	HR (95% CI)	Wald test	*P* value
**MM (*n* = 163)**				
*NOTCH1*	4.924	137.5 (8.6–2200)	12.12	0.0004986
*ZRSR2*	4.224	68.33 (6.2–750)	11.90	0.0005624
*CDKN1B*	2.049	7.759 (2.3–26)	10.70	0.0010690
*BIRC3*	2.077	7.977 (1.8–35)	7.63	0.0057340
*CHD8*	1.948	7.012 (1.6–31)	6.46	0.0110600
*PCLO*	1.031	2.803 (1.1–7.5)	4.23	0.0396200
**AL (*n* = 121)**				
*CREBBP*	2.030	7.611 (2.3–25)	10.88	0.0009738
*LTB*	3.576	35.74 (3.7–340)	9.59	0.0019540
*DNMT3A*	2.064	7.875 (1.8–34)	7.66	0.0056610
*PRKD2*	2.854	17.36 (2.1–140)	7.07	0.0078410
*ARID1B*	1.242	3.463 (1.2–9.8)	5.45	0.0196200
*SYNE1*	0.727	2.07 (1.0–4.2)	4.15	0.0415200
**POEMS (*n* = 67)**				
*JAK2*	2.343	10.41 (2.2–49)	8.73	0.0031360
*CCND1*	2.508	12.28 (1.4–110)	5.24	0.0221000
*ATR*	2.291	9.883 (1.2–82)	4.49	0.0340300
*PTPRD*	2.291	9.883 (1.2–82)	4.49	0.0340300

*Note*: CH, clonal hematopoietic; HR, hazard ratio; CI, confidence interval.

### BMF subgroups of MM demonstrated distinct PFS despite similar age at diagnosis

**Figure 4:**
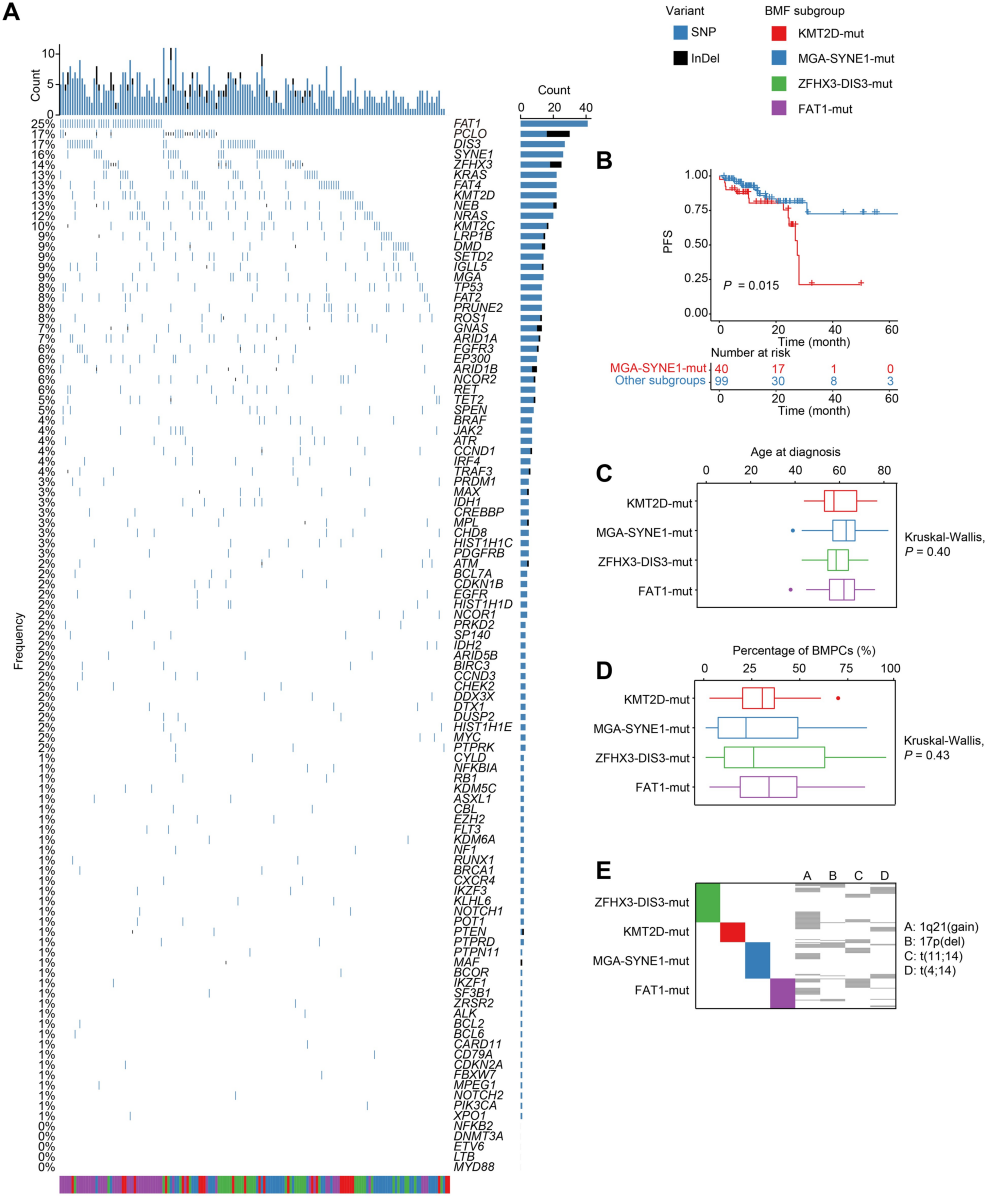
Clinical characteristics of BMF subgroups of MM **A**. Waterfall plot showing lymphoid and myeloid CH mutations and myeloma driver gene mutations in MM (*n* = 163). **B**. Kaplan–Meier curves showing the PFS of patients with MM in the MGA-SYNE1-mut subgroup. **C**. Age at diagnosis of patients with MM in different BMF subgroups. **D**. Percentages of BMPCs of patients with MM in different BMF subgroups. **E**. Associations between BMF subgroups and variants, including 1q21(gain), t(11;14) (IgH/CCND1), t(4;14) (IgH/FGFR3), and 17p(del). PFS, progression-free survival; SNP, single nucleotide polymorphism; InDel, insertion and deletion.

We hypothesized that the BMF subgroups in each of the PCD subtypes may indicate clinical performance. To compare our findings with existing knowledge, we examined the BMF subgroups within MM, the most extensively studied PCD subtype. The mutation rates of *KRAS*, *NRAS*, *FGFR3*, *BRAF*, *TP53*, *ATM*, and *CCND1* were close to those reported by the Multiple Myeloma Research Foundation (MMRF) CoMMpass study [31] and other previous studies [9–11] (**Figure 4**A). Then, we validated the co-occurrence of single nucleotide variants (SNVs) and cytogenetic variants: *DIS3* and 1q21(gain), *DIS3* and t(4;14) (IgH/FGFR3), *TP53* and 17p(del), and *CCND1* and t(11;14) (IgH/CCND1) (Figure S2A) [9]. Also, we found no co-occurrence among these cytogenetic abnormalities and the BMF subgroups (Figure S2B). The prognostic value of International Staging System (ISS) stage III was valid in our MM cohort, which showed the reliability of our clinical data (Figure S3A).

Among the BMF subgroups, the MGA-SYNE1-mut subgroup showed significantly worse PFS (*P* = 0.015) (Figure 4B, Figure S3B). These subgroups were not associated with the patient’s age at diagnosis (Figure 4C) or the proportion of plasma cells in the bone marrow (Figure 4D). Unlike SNVs, BMF subgroups were not prominently associated with 1q21(gain), t(11;14) (IgH/CCND1), t(4;14) (IgH/FGFR3), or 17p(del) (Figure 4E).

These results demonstrated that the mutational profile of MM is consistent with that in previous reports and highlighted the dismal PFS of patients in the MGA-SYNE1-mut subgroup. This consistency supported our further analysis of other PCD subtypes.

### BMF subgroups of AL showed distinct PFS, plasma cell burden, and serum differential free light chain levels

**Figure 5:**
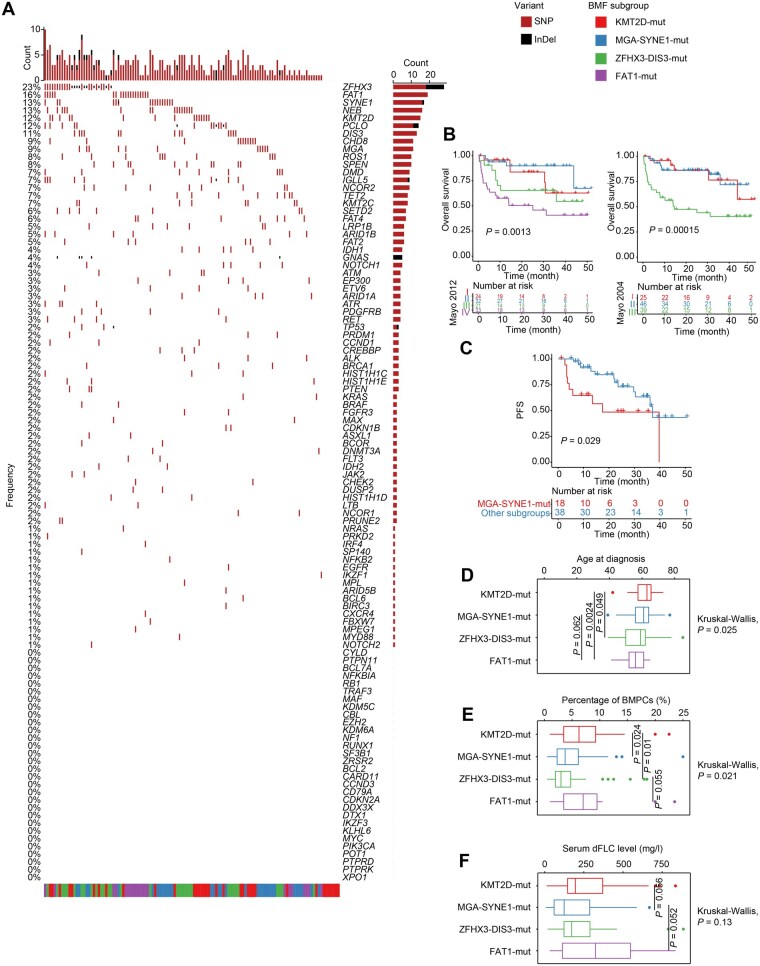
Clinical characteristics of BMF subgroups of AL **A**. Waterfall plot showing lymphoid and myeloid CH mutations and myeloma driver gene mutations in AL (*n* = 121). **B**. Kaplan–Meier curves showing the overall survival of patients at different stages of the Mayo 2012 / Mayo 2004 staging systems. **C**. Kaplan–Meier curves showing the PFS of patients with AL in the MGA-SYNE1-mut subgroup. **D**. Age at diagnosis of patients with AL in different BMF subgroups. **E**. Percentages of BMPCs of patients with AL in different BMF subgroups. **F**. Serum dFLC levels of patients with AL in different BMF subgroups. dFLC, differential free light chain.

Using the MM results as a reference, we then studied the clinical relevance of BMF subgroups in AL. The most recurrent variants in AL included those in *ZFHX3*, *FAT1*, *KMT2D*, *DIS3*, *IGLL5*, *SETD2*, and *FAT4*, most of which have been reported previously (**Figure 5**A) [2,15–18]. We confirmed that the Mayo 2012 and Mayo 2004 staging systems effectively classified a patient’s risk of disease progression (Figure 5B) and that t(11;14) (IgH/CCND1) predicted dismal prognoses (Figure S3C) [32].

Similar to the result in MM, patients at Mayo stages I and II of the MGA-SYNE1-mut subgroup exhibited early deterioration (*P* = 0.029), whereas those of the ZFHX3-DIS3-mut subgroup were free from disease progression until approximately the 40th month (Figure 5C, Figure S3D). This difference in PFS was not found among patients at the stages III and IV in the Mayo 2012 staging system, probably due to their severe cardiac dysfunction.

The patients in the different BMF subgroups also differed in age at diagnosis, the burden of plasma cells in the bone marrow, and serum differential free light chain (dFLC) levels. Remarkably, patients in the FAT1-mut subgroup were younger at diagnosis than those in the ZFHX3-DIS3-mut, MGA-SYNE1-mut, and especially KMT2D-mut subgroups (*P* = 0.025) (Figure 5D). The FAT1-mut subgroup also exhibited a higher BMPC burden (*P* = 0.021) (Figure 5E) and higher serum dFLC levels (*P* = 0.13) (Figure 5F) than the other subgroups. Specifically, most of the patients with AL in the FAT1-mut subgroup were diagnosed before 60 years old, possessing an over 5% median percentage of BMPCs and a median serum dFLC level of above 300 mg/l with great variance. These results suggest that the FAT1-mut subgroup might be associated with a worse prognosis.

Collectively, the patients with AL in the MGA-SYNE1-mut subgroup were susceptible to early disease progression. In contrast, those in the FAT1-mut subgroup were younger patients with higher plasma cell burden and elevated serum dFLC levels.

### BMF subgroups of POEMS showed differences in the age at diagnosis and serum VEGF levels

**Figure 6:**
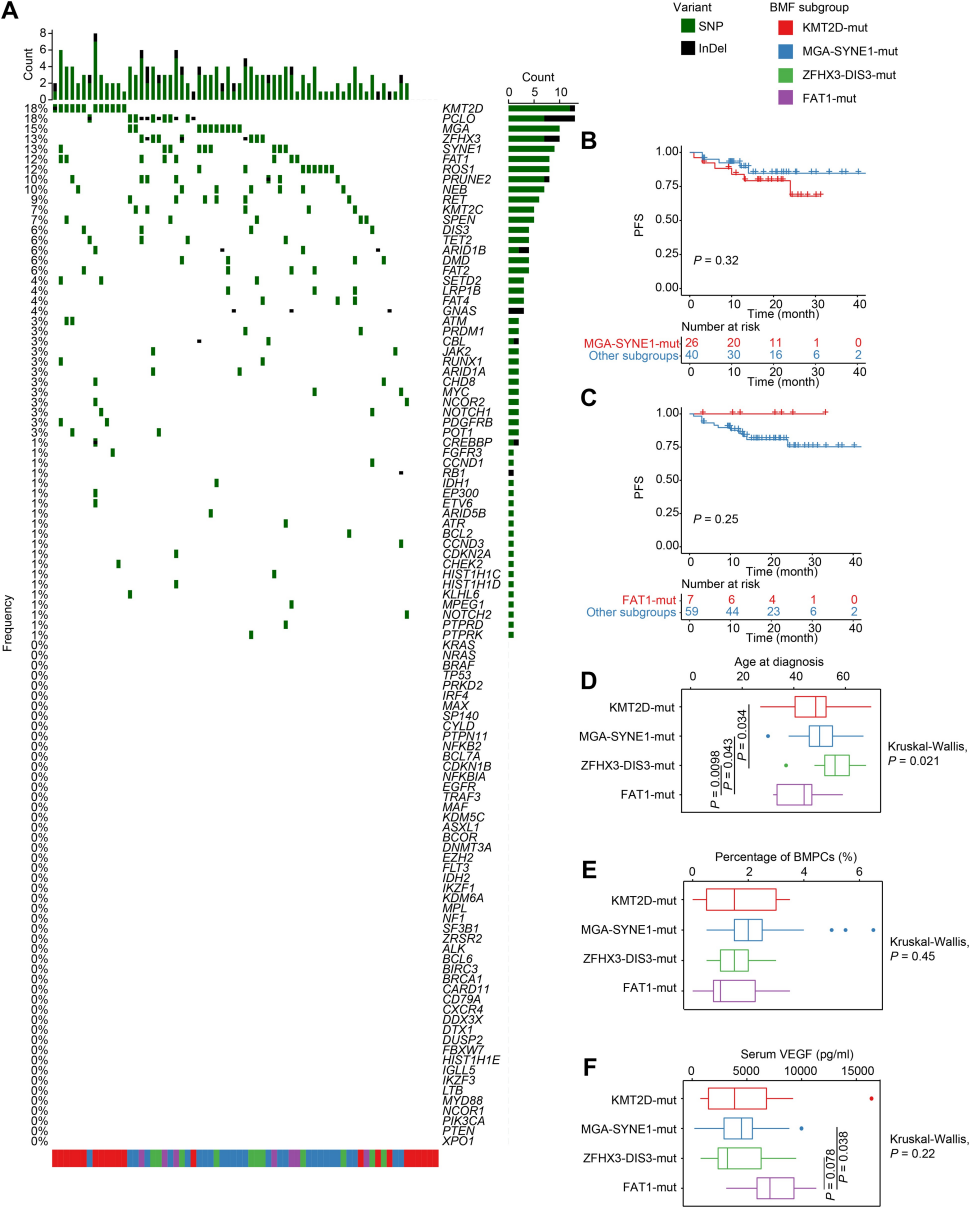
Clinical characteristics of BMF subgroups of POEMS **A**. Waterfall plot showing lymphoid and myeloid CH mutations and myeloma driver gene mutations in POEMS (*n* = 67). **B**. Kaplan–Meier curves showing the PFS of patients with POEMS in the MGA-SYNE1-mut subgroups. **C**. Kaplan–Meier curves showing the PFS of patients with POEMS in the FAT1-mut subgroups. **D**. Age at diagnosis of patients with POEMS in different BMF subgroups. **E**. Percentages of BMPCs of patients with POEMS in different BMF subgroups. **F**. Serum VEGF levels of patients with POEMS in different BMF subgroups. VEGF, vascular endothelial growth factor.

Lastly, we explored the roles of the BMF subgroups in POEMS, a rare type of PCD. The most recurrent CH mutations in POEMS were those in *KMT2D*, *PCLO*, *MGA*, *ZFHX3*, *SYNE1*, *FAT1*, and *ROS1* (**Figure 6**A).

Limited by the indolent nature of POEMS, patients with POEMS in the MGA-SYNE1-mut subgroup showed a non-significant downtrend (Figure 6B, Figure S3E), whereas those in the FAT1-mut subgroup exhibited slightly stable disease when compared with the other patients (Figure 6C). The FAT1-mut subgroup was composed of significantly younger patients at diagnosis (*P* = 0.021) (Figure 6D), with similar plasma cell burden in their bone marrow (Figure 6E) and higher serum VEGF levels (Figure 6F) than those in the other subgroups. In contrast, the patients in the ZFHX3-DIS3-mut subgroup were significantly more elderly at diagnosis (Figure 6D) and had lower levels of serum VEGF (Figure 6F) than those in the other subgroups. Patients in the FAT1-mut subgroup were characterized by a median age of ∼ 40 years old and a median serum VEGF level of over 6000 pg/ml.

These results showed that the FAT1-mut subgroup included patients younger at diagnosis with higher serum VEGF levels, whereas those in the ZFHX3-DIS3-mut subgroup showed the opposite trend.

### BMF subgroups were defined by CH mutations with significance in MGUS remaining to be determined

Additionally, we found CH mutations and analyzed BMF subgroups among the patients with MGUS, the known premalignant status of MM. The dominant CH mutations in MGUS included those in *KMT2D*, *FAT1*, *MGA*, and *DIS3*, whereas the subclonal mutations were in *ASXL1*, *HIST1H1C*, and *MAF* (Figure 1F), overlapping with the other PCD subtypes (Figure 2A). The most recurrent mutations in MGUS included those in *KMT2D*, *FAT1*, *PCLO*, *PRDM1*, *DIS3*, *ZFHX3*, and *MAF* (Figure S4). These findings suggest that MGUS is an entity in the spectrum of PCDs as evidenced by the similarity of its CH mutations to the other PCD subtypes.

## Discussion

This study has provided insights into the clinical relevance of lymphoid and myeloid CH mutations in a relatively large sample of PCDs. Our data are valuable because we have aimed to explain the shared pathogenesis of PCDs, especially that of rare subtypes like AL and POEMS. Meanwhile, we have explored the CH mutations that play an emerging role in hematology, which has provided a new perspective on the long-standing problems regarding PCDs [33].

Based on the assumption that CH mutations are enriched in monoclonal plasma cells, we sequenced the BMPCs of patients with PCDs without removing the mutations in peripheral blood lineages and found abundant CH mutations in BMPCs. Previous studies revealed similar VAFs of CH mutations (0.45 < VAF < 0.55) in pathogenic cells in Erdheim–Chester disease [23,34], bone marrow cells of patients with myelodysplastic/myeloproliferative neoplasms [35], and tumor cells of Epstein–Barr virus-positive diffuse large B cell lymphoma [36]. Our results provide new insights into CH mutations in the context of PCDs, an important category of hematological malignancies.

Mechanistically, CH mutations have functional roles in chromatin remodeling, epigenetic modifications, DNA damage repair, cell growth, and proliferation, which provides hints on how they potentially give rise to PCDs. Similarly, previous studies observed that mutations in epigenetic modifiers occurred in premalignant stages or clonal plasma cells [2]. The progression from MGUS or SMM to MM involved mutations in *KMT2D*, *DIS3*, and *KDM1B* during the early transition [37]. A study on the epigenetic modifier mutations in MM observed mutations in *KDM6A*, *ARID2*, *HIST1H1E*, and *HIST1H1C* in BMPCs [38]. A mutation in *EP300* with a similar VAF was found in plasma cell clones in a case report on POEMS syndrome [39]. In addition to these direct observations, rare germline variants in *EP300*, *CDKN2A*, *ARID1A*, and *DIS3* have been associated with an increased risk of MM, which implies that they may participate in disease initiation [40].

Remarkably, we have found a subgroup of PCDs with *FAT1* mutations showing increased serum levels of VEGF and dFLC, which might answer the crucial question of what causes VEGF production in POEMS [19]. FAT1, an integral membrane protein regulating Wnt signaling that negatively regulates amyloidogenic processing in Alzheimer’s disease [41], has critical roles in solid tumors and has been demonstrated to be highly mutated in high-risk chronic lymphocytic leukemia [42]. However, the mechanisms that links *FAT1* mutations to the patient’s age at diagnosis and serum levels of VEGF or dFLC remain unknown. Given the large size of the *FAT1* gene, mutations in it are more likely to represent an accumulation of somatic mutations than driver mutations.

Our study has several issues with the methodology that might limit the interpretation and extrapolation of the results. Firstly, we did not use paired peripheral blood mononuclear cells as controls for the BMPC samples, which prevented the exclusion of somatic mutations that also existed in peripheral blood cell lineages [22] but retained some germline mutations that appeared in both peripheral blood cells and BMPCs [43]. Moreover, BMPCs were not isolated with flow cytometry as the surface markers of AL and POEMS are unknown; therefore, our samples are likely to include both malignant and normal BMPCs. Those BMPCs were sequenced by a limited panel of 103 gene loci, which partially accounts for the lack of observed co-occurrence between these cytogenetic abnormalities and the BMF subgroups in MM.

Another caveat of our study is the difficulty of reaching solid conclusions based on this treatment data for several reasons. First, the number of patients who fall in each category of treatment response is very low (usually < 5). Second, these data are from a retrospective patient database but not a prospective cohort study (Table S4).

One objective of future research is to determine the origin of these mutations in the premalignant stages of PCDs, including hematopoietic cells, lymphoid progenitor cells, B lymphocytes, and normal plasma cells. Capturing CH mutations within these cell subsets in PCD patients [22] will require single-cell technology, which has been instrumental in studying plasma cell fate programming in both health and disease [44]. For example, there are studies on the heterogeneity of B cell subsets in MM and its premalignant stages [45] and the association between B cell lineage and the malignancy of MM and AL [46].

In conclusion, CH mutations in plasma cells partially explained the shared pathogenesis of MM associated with clinical manifestations of MM, AL, POEMS, and MGUS. A subgroup characterized by *FAT1* mutations included younger AL patients with elevated serum dFLC levels and younger POEMS patients with higher serum VEGF levels. Another subgroup with *MGA* or *SYNE1* mutations showed unfavorable PFS, which was true for patients with MM, AL, and POEMS. Future clinical practice may identify patients of these specific subgroups by testing CH mutations in BMPCs.

## Materials and methods

### Patient sample preparation

Bone marrow aspirate samples were donated by 163 patients with MM, 121 patients with AL, 67 patients with POEMS, 13 patients with MGUS, and 20 healthy controls, all of whom provided informed consent (see Ethical statement). The inclusion criteria were according to the current guidelines and recommendations [3,5,6].

A 9-ml bone marrow aspirate was diluted with 9 ml of phosphate-buffered saline, and the lymphocytes were separated using a lymphocyte separation medium (Catalog No. 07811, STEMCELL Technologies, Vancouver, Canada). Magnetic beads conjugated to CD138 antibodies (Catalog No. 130-135-361, Miltenyi Biotec, Bergisch Gladbach, Germany) were used to enrich the CD138^+^ BMPCs from the isolated lymphocytes.

### Detection of cytogenetic abnormalities

A hypotonic potassium chloride solution (0.075 M) and a Carnoy’s solution [3:1 (v/v) methanol/acetic acid] were used to wash the isolated lymphocytes. The cell pellet was resuspended in Carnoy’s solution and centrifuged again to wash the cells. Then, interphase fluorescence *in situ* hybridization (FISH) with probe kits (Abbott Molecular, Des Plains, IL) was used to detect cytogenetic abnormalities in the BMPCs. The thresholds for cytogenetic abnormalities were set according to the recommendation of European Myeloma Network: t(11;14) > 10%, 1p21(gain) > 20%, 17p(del) > 20%, and t(4;14) > 10% [3]. Probe hybridization was observed using an OLYMPUS BX51 objective fluorescence microscope (Catalog No. WS-BX51-0169, OLYMPUS, Tokyo, Japan).

### Targeted gene sequencing

DNA was extracted using a DNeasy Blood and Tissue Kit (Catalog No. 69504, Qiagen, Valencia, CA). DNA quality was validated via agarose gel electrophoresis and quantified using a Qubit DNA assay kit on a Qubit 2.0 Fluorometer (Catalog No. Q32866, Life Technologies, Carlsbad, CA).

The list of lymphoid and myeloid CH mutations was obtained from a recent cohort study of clonal hematopoiesis of intermediate potential [21]. The list of myeloma driver genes was collected from the IntOGen database [47]. These BMPC DNA libraries were analyzed through targeted gene sequencing of a panel of lymphoid CH mutations, myeloid CH mutations, and myeloma driver genes (Table S1). Then, the DNA libraries were sequenced with an Illumina HiSeq 4000 platform (Catalog No. SY-401-4001, Illumina, San Diego, CA) at a mean sequencing depth of 1000×.

### Filtering and selection of somatic variants

The valid sequences in the FASTQ files were mapped to a reference genome (University of California, Santa Cruz hg38) using BWA software [48] after performing quality control with fastp software [49]. SNVs were detected using the Genome Analysis Toolkit (GATK, v4) software [50] and then annotated using the ANNOtate VARiation (ANNOVAR) software [51]. The 20 healthy controls were used to create a panel of normal (PoN) that includes mutations that should be excluded from the diseased samples. The single nucleotide polymorphism database (dbSNP) was also used by the GATK (v4) pipeline to exclude potential single nucleotide polymorphisms (SNPs).

Since no peripheral blood cell samples were used as controls, we defined the likely somatic variants [including SNVs and insertions and deletions (InDels)] as those that are rare in the healthy population, potentially pathogenic, and among the known CH mutation genes or myeloma driver genes. The exclusion criteria for variants: sequencing depth < 50 (mean sequencing depth = 1000); VAF < 0.02; synonymous SNVs; genomicSuperDups = TRUE; population frequency: ExAC ALL > 0.01 or 1000 Genomes 2015.08 ALL > 0.01; or pathogenicity prediction: CADD pred < 15 or SIFT score > 0.05. Among the likely somatic variants (including non-driver and driver mutations), dominant clonal variants were those with a VAF between 0.45 and 0.55, while subclonal variants were those with a VAF lower than 0.45 [52].

### BMF

We performed non-supervised BMF, a machine-learning algorithm developed to study the genetic heterogeneity of SMM, a disease in the PCD spectrum [53]. It is suitable for analyzing sparse, non-negative, and binarized data, such as gene mutations. We included all SNVs in the analysis regardless of their frequency of occurrence. The number of subgroups (*k*) was set as 4, and the number of iterations as 50; other parameters were set to the default values.

### Statistical analysis and visualization

R (v4.0.3) and Python (v3.7) were used for statistical analysis and visualization. Statistical tests, such as the Kruskal–Wallis test, were two-sided, and the data distribution was assumed to be normal without being tested. *P* < 0.05 was considered statistically significant. Survival curves were generated using the Kaplan–Meier method, and PFS was defined as the time between the dates of diagnosis and the last follow-up or relapse.

## Ethical statement

The written informed consents were obtained from the participating patients. This study was approved by the Ethics Committee of the Peking Union Medical Hospital, China (Approval No. K5339).

## Code availability

The code and analysis data have been submitted to BioCode at the National Genomics Data Center (NGDC), China National Center for Bioinformation (CNCB) (BioCode: BT007606), which are publicly accessible at https://ngdc.cncb.ac.cn/biocode/tool/BT7606 as “TGS_PCDs”.

## Supplementary Material

qzaf027_Supplementary_Data

## Data Availability

The targeted gene sequencing data have been deposited in the Genome Sequence Archive for Human [54] at the NGDC, CNCB (GSA-Human: HRA009164), and are publicly accessible at https://ngdc.cncb.ac.cn/gsa-human.

## CRediT author statement

**Xuezhu Wang:** Methodology, Formal analysis, Writing – original draft. **Liping Zuo:** Investigation. **Yanying Yu:** Investigation. **Xinyi Xiong:** Methodology. **Jian Xu:** Resources. **Bing Qiao:** Resources. **Jia Chen:** Resources. **Hao Cai:** Methodology. **Qi Yan:** Writing – review & editing. **Hongxiao Han:** Resources. **Xin-xin Cao:** Resources. **Jun Deng:** Resources. **Chunyan Sun:** Conceptualization, Supervision. **Jian Li:** Conceptualization, Supervision. All authors have read and approved the final manuscript.
