# Supplementary material for: Clonal Hematopoietic Mutations in Plasma Cell Disorders: Clinical Subgroups and Shared Pathogenesis
Source: Genomics Proteomics Bioinformatics. 2025 Mar 27;23(2):qzaf027. doi: 10.1093/gpbjnl/qzaf027 (PMC12342758; doi:10.1093/gpbjnl/qzaf027)
Supplement: qzaf027_Supplementary_Data [file qzaf027_supplementary_data.zip › supplementary material captions.docx]

**Supplementary material**

**Figure S1 BMF analysis of lymphoid and myeloid CH mutations**

**A.** BMF analysis of lymphoid and myeloid CH mutations, and myeloma driver mutations (k = 2). **B.** BMF analysis of lymphoid and myeloid CH mutations, and myeloma driver mutations (k = 3). **C.** BMF analysis of lymphoid and myeloid CH mutations, and myeloma driver mutations (k = 4). **D.** BMF analysis of lymphoid and myeloid CH mutations, and myeloma driver mutations (k = 5). **E.** BMF analysis of lymphoid and myeloid CH mutations, and myeloma driver mutations (k = 6). **F.** BMF analysis of lymphoid and myeloid CH mutations, and myeloma driver mutations (k = 7).

**Figure S2 Co-occurrence among genetic variations**

**A.** Co-occurrence among genetic variations in MM. SNVs includes *CCND1*, *DIS3*, *FGFR3*, *KRAS*, *NRAS*, and *TP53*. Cytogenetic abnormalities includes 1q21(gain), 17p(del), t(11;14) (IgH/CCND1), and t(4;14) (IgH/FGFR3). **B.** Co-occurrence among cytogenetic abnormalities and the BMF subgroups.

**Figure S3 Overall survival of patients in different BMF subgroups**

**A.** Kaplan–Meier curves demonstrating overall survival of patients with MM with different ISS staging. **B.** Kaplan–Meier curves demonstrating progression-free survival of patients with MM in different BMF subgroups. **C.** Kaplan–Meier curves demonstrating overall survival of patients with AL with or without t(11;14). **D.** Kaplan–Meier curves demonstrating progression-free survival of patients with AL in different BMF subgroups. **E.** Kaplan–Meier curves demonstrating progression-free survival of patients with POEMS in different BMF subgroups. ISS, International Staging System.

**Figure S4 Lymphoid and myeloid CH mutations and myeloma driver mutations**

Waterfall plot showing lymphoid and myeloid CH mutations, and myeloma driver mutations in monoclonal gammopathy of undetermined significance.

**Table S1 The full list of clonal hematopoietic mutations observed in this study**

**Table S2 The variant allele frequency of clonal hematopoietic mutations in each sample**

**Table S3 The complete list of multivariate Cox proportional hazards model**

**Table S4 Treatment response data of the patients in different binary matrix factorization subgroups**
