# Supplementary figures and images for: Clonal Hematopoietic Mutations in Plasma Cell Disorders: Clinical Subgroups and Shared Pathogenesis
Source: Genomics Proteomics Bioinformatics. 2025 Mar 27;23(2):qzaf027. doi: 10.1093/gpbjnl/qzaf027 (PMC12342758; doi:10.1093/gpbjnl/qzaf027)

A

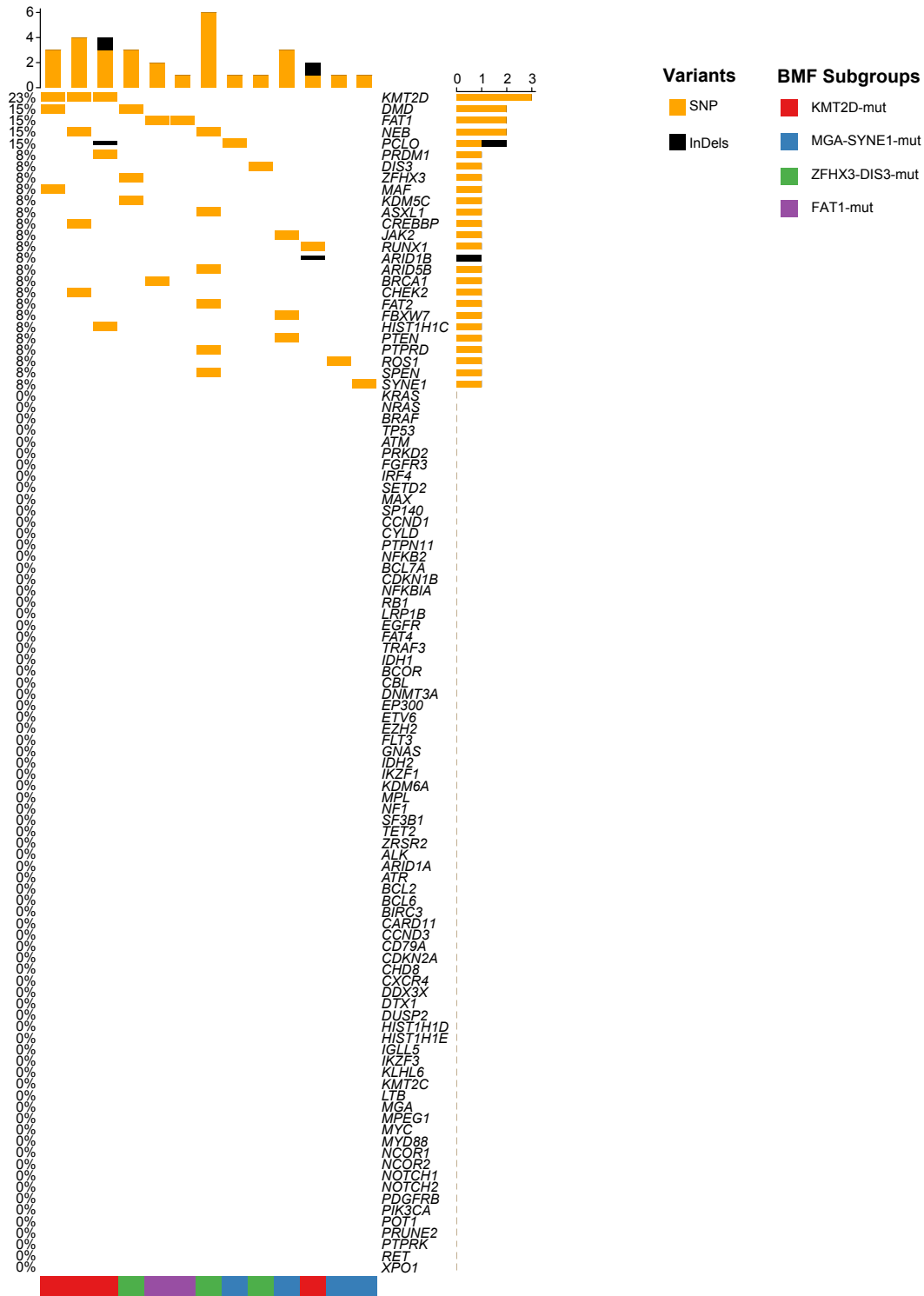

Supplement: qzaf027_Supplementary_Data [file qzaf027_supplementary_data.zip › figS4.pdf]

**A**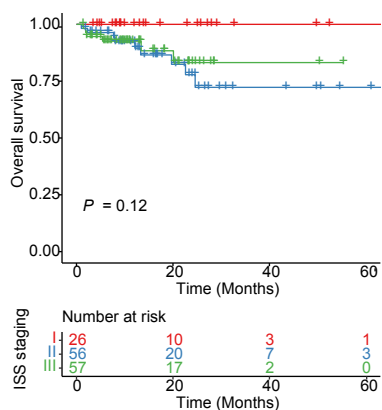**B**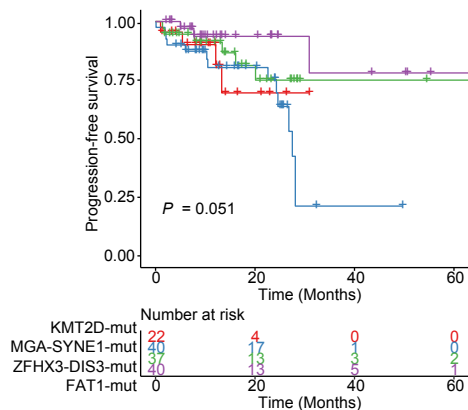**C**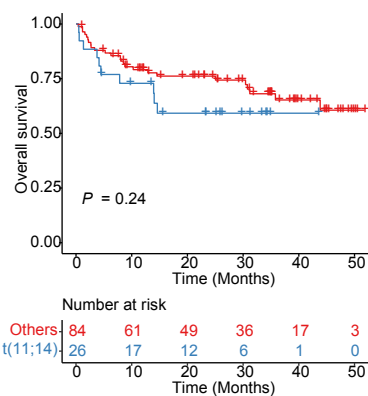**D**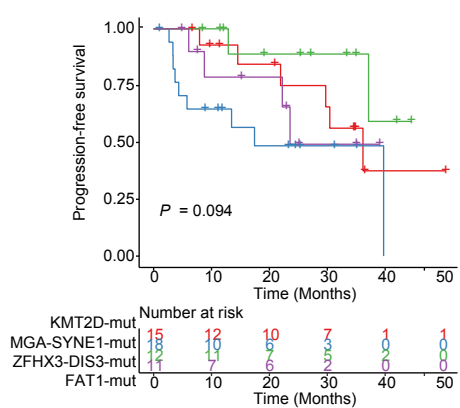**E**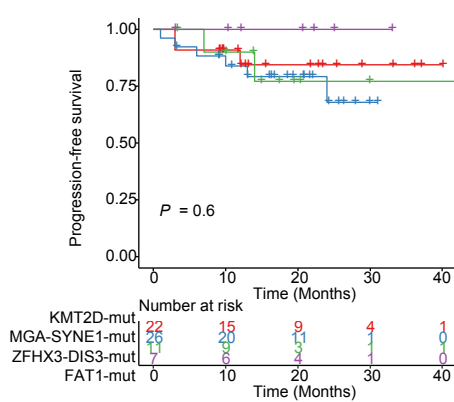

Supplement: qzaf027_Supplementary_Data [file qzaf027_supplementary_data.zip › figS3.pdf]

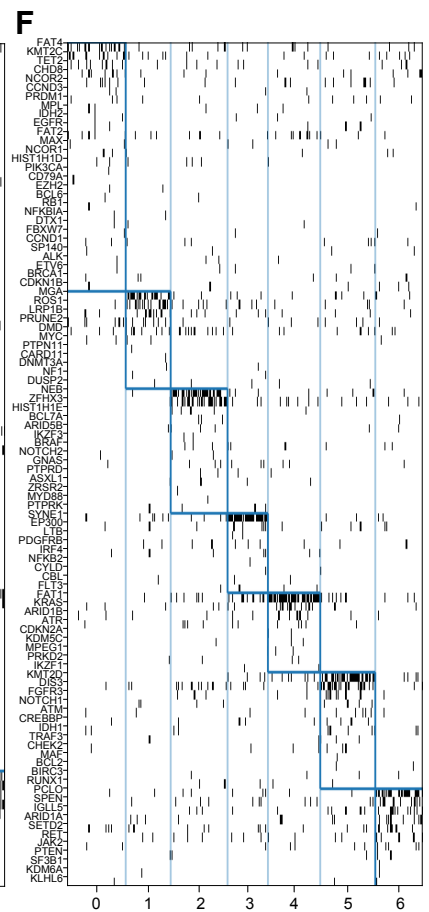

Supplement: qzaf027_Supplementary_Data [file qzaf027_supplementary_data.zip › figS1.pdf]

**A**

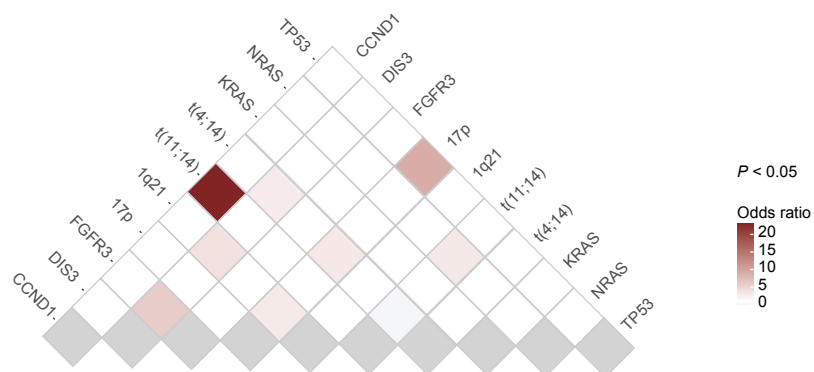

# B

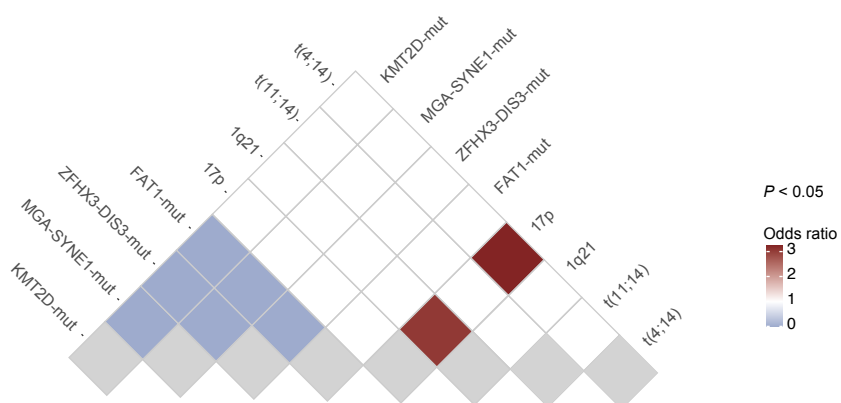

Supplement: qzaf027_Supplementary_Data [file qzaf027_supplementary_data.zip › figS2.pdf]
